# Characterization of a Soluble B7-H3 (sB7-H3) Spliced from the Intron and Analysis of sB7-H3 in the Sera of Patients with Hepatocellular Carcinoma

**DOI:** 10.1371/journal.pone.0076965

**Published:** 2013-10-23

**Authors:** Weiwei Chen, Peixin Liu, Yedong Wang, Weimin Nie, Zhiwei Li, Wen Xu, Fengyi Li, Zhiping Zhou, Min Zhao, Henggui Liu

**Affiliations:** 1 State Key Laboratory of Veterinary Biotechnology, Harbin Veterinary Research Institute, the Chinese Academy of Agricultural Sciences, Harbin, China; 2 Treatment and Research Center for Infectious Diseases, 302 Hospital of P.L.A., Beijing, China; University of Alabama at Birmingham, United States of America

## Abstract

B7-H3 is a recently discovered member of the B7 superfamily molecules and has been found to play a negative role in T cell responses. In this study, we identified a new B7-H3 isoform that is produced by alternative splicing from the forth intron of B7-H3 and encodes the sB7-H3 protein. Protein sequence analysis showed that sB7-H3 contains an additional four amino acids, encoded by the intron sequence, at the C-terminus compared to the ectodomain of 2Ig-B7-H3. We further found that this spliced sB7-H3 plays a negative regulatory role in T cell responses and serum sB7-H3 is higher in patients with hepatocellular carcinoma (HCC) than in healthy donors. Furthermore, we found that the expression of the spliced *sb7-h3* gene is higher in carcinoma and peritumor tissues than in PBMCs of both healthy controls and patients, indicating that the high level of serum sB7-H3 in patients with HCC is caused by the increased expression of this newly discovered spliced sB7-H3 isoform in carcinoma and peritumor tissues.

## Introduction

An optimal T cell response requires two signals which are delivered through the antigen-specific T cell receptor (TCR) and accessory molecules. A cohort of the B7 superfamily ligands are important accessorial molecules and play co-stimulatory or co-inhibitory roles in T cell responses. They have been found to be an essential contributor to T-cell activation and tolerance [1]–[4]. Recent studies have demonstrated that some inhibitory B7 family ligands, such as B7-H1, B7-DC, B7-H3 and B7-H4, are highly expressed in a wide spectrum of human cancers, and their expression levels are correlated with patients' clinicopathological features. Moreover, increasing expression of inhibitory B7 molecules may reflect the poor prognosis for the patients with carcinoma [5]–[7]. Therefore, it has been proposed that cancers evade immune supervision by modulating the expression of inhibitory B7 molecules.

B7-H3 was recently identified as a member of the B7 superfamily [8]. It is broadly expressed in human and murine tissues at the RNA level, however, its protein expression is relatively rare [8]. The role of B7-H3 in the immune response still remains controversial. It was first reported to be a positive regulator for T cell response [8], [9], but later research showed its ability to negatively regulate T cell response, inhibiting T cell proliferation and cytokine secretion [10]–[13]. Many studies have demonstrated that B7-H3 expression is aberrant on a variety of tumor tissues, with high expression correlated to the progression and poor prognosis for patients with cancer [14]–[19], suggesting that B7-H3 expression level may be used as a biomarker for cancers [20], [21]. Currently, there are two known isoforms of B7-H3 in humans, 2Ig- and 4Ig-B7-H3. Apart from the membrane B7-H3 proteins, a serum soluble B7-H3 was also reported in humans by Dr. Zhang [22]. Further investigation revealed that serum sB7-H3 is significantly higher in patients with carcinoma than in healthy donors [23]. However, there is no direct evidence to show how sB7-H3 is produced in humans and the reason for the higher levels of sB7-H3 in patient sera.

HCC is the fifth most common cancer and the third most common cause of cancer death worldwide [24]. Due to the absence of a sensitive diagnostic biomarker, most patients with HCC are diagnosed in the late stages and present with disease too advanced for curative treatment. sB7-H3 has recently found to be a biomarker for cancers [23], [25]. However, it remains unclear whether expression of sB7-H3 is abnormal in the sera of patients with HCC. Here, we identified a new B7-H3 isoform and further investigated its characteristics and clinical implication of serum sB7-H3 in patients with HCC.

## Materials and Methods

### Study subjects

50 HCC patients (42 males, 8 females; median age, 37.2 years) hospitalized in Beijing 302 Hospital from December 2010 to August 2012 along with 60 gender- and age-matched healthy donors were enrolled for the study. Plasma samples and PBMCs were obtained from all enrolled subjects. Diagnosis of HCC was based on the criteria of the European Association for the Study of the Liver [26]. To analyze the spliced sB7-H3 in hepatoma and peritumor tissues, 25 of 50 HCC patients who had undergone surgical resection were enrolled. Solid hepatoma tissues and peritumor tissues were excised about 1 cm but no more than 3 cm away from the invasive tumor margin, as previously reported [27], [28]; samples were snap-frozen in liquid nitrogen for RNA extraction and subsequent cDNA synthesis. To analyze the influence of hepatoma on the level of spliced sB7-H3 in serum, 10 HCC patients who had undergone surgical resection were enrolled, and their sera obtained before resection and 2 weeks after were analyzed for spliced sB7-H3 by sandwich ELISA assay. Our protocol was approved by the Ethics Committee of Beijing 302 Hospital under the permit number 2012011D and written informed consent was obtained from each subject.

### PBMC Isolation and gene cloning

PBMCs from patients and healthy donors were isolated by Ficoll-Hypaque gradient centrifugation as previously reported [29]. Total RNA of PBMCs was isolated and cDNA was synthesized using RNeasy plus mini kit (QIAGEN) and PrimeScript 1^st^ strand cDNA synthesis kit (TaKaRa), respectively. To clone human B7-H3, U937 cells were subjected to RNA extraction and cDNA synthesis using the specific primer pair, B7-H3-forward: 5-′CTCACAGGAAGATGCTGCGTC-3′ and B7-H3-reverse: 5′-CGGAAGGCATCAGAACGTCT-3′. The PCR was performed under the following conditions: 95°C for 3 min followed by 30 cycles of denaturing at 94°C for 30 sec, annealing at 60°C for 30 sec and extension at 72°C for 2 min. The PCR products were inserted into pMD18-T vector and sequenced. To check the existence of spliced sB7-H3 in healthy donors and hepatoma cell line, a special primer pair, sB7-H3-F: 5′- GTTGCTTTGCTTAAATGTCCC-3′ and sB7-H3-R: 5′-GGAGTCCTTGAGGGAGGAAC-3′, which were resided at the position of the spliced intron 4 and exon 10 respectively, were designed to specifically amplify a 247 bp-length of sequence from spliced sB7-H3.

### Real-time PCR assay

Total RNA and cDNA of the samples were isolated and synthesized as described above. Quantitative real-time PCR assay was performed using specific primer pairs for spliced B7-H3 (forward: 5′- CCCACAGGTTGCTTTGCTTAA-3′ and reverse: 5′-GCAGACCCCTGGAGAACCA-3′) and β-actin (Forward: 5′-CAGCTCACCATGGATGATGATATC-3′ and reverse: 5′-AAGCCGGCCTTGCACAT-3′) in the presence of SYBR Green. Amplification was performed under the following conditions: holding at 95°C for 3 min followed by 40 cycles of denaturing at 95°C for 20 sec, annealing at 58°C for 20 sec and extension at 72°C for 20 sec. Gene expression was quantified using the comparative C_T_ method as described in the ABI7700 User Bulletin 2.

### Sandwich ELISA assay

To quantify the sB7-H3 in sera, a sandwich ELISA assay was established using a mouse anti-human B7-H3 monoclonal antibody (Santa cruz biotechnology) as capture antibody and a rabbit anti-human B7-H3 polyclonal antibody (Santa cruz biotechnology) as detection antibody. 50 µl serum was added to each well of the ELISA plate precoated with 1 µg/ml capture antibody overnight at 4°C and blocked with 1% BSA at 37°C for 2 h. The spliced sB7-H3 was detected with the detection antibody and HRP-conjugated goat anti-rabbit immunoglobulin antibody. Plates were washed four times with PBST, after which TMB substrate was added to detect for the presence of spliced sB7-H3. The relative concentration of spliced sB7-H3 in sera was determined by optical density (OD) reading at 450 nm.

### Generation of Ig-fusion proteins

The spliced sB7-H3 was fused with rabbit Fc in one ORF and inserted into the pcDNA3.1(+) vector to generate pc-sB7-H3-Fc. A recombinant vector expressing rabbit Fc fused with human PD-1 signal peptide was also generated (pc-Fc). Soluble proteins were produced by transfecting pc-sB7-H3-Fc or pc-Fc into 293T cells and purified as previously reported [30], [31]. The concentration of purified sB7-H3-Fc and Fc were determined with the rabbit IgG ELISA quantification kit (Bethyl).

### T cell proliferation and cytokine assay

T cells were isolated with anti-CD3 dynabeads and labeled with 2.5 µM CFSE, as previously reported [30]. 1×10^5^ CD3^+^ T cells/well were seeded to 96-well flat-bottomed plates precoated with 1 µg/ml anti-human CD3 (OKT3) plus either 10 µg/ml spliced sB7-H3-Fc or Fc, as previously reported [30], [32]. After culturing for 72 h, T cell proliferation was analyzed based on CFSE-dilution by FACS assay. Cytokines in the culture supernatant were detected with IL-2 and IFN-γ multiplex kits (Bender MedSystems) and analyzed by FlowCytomixPro software.

### Statistical analysis

All statistical analyses were performed using the paired or independent student's *t*-test. P values less than 0.05 were considered statistically significant. Data were presented as mean values ± the standard error of the mean (SEM) of at least three independent experiments.

## Results

### Identification of spliced sB7-H3

**Figure 1:**
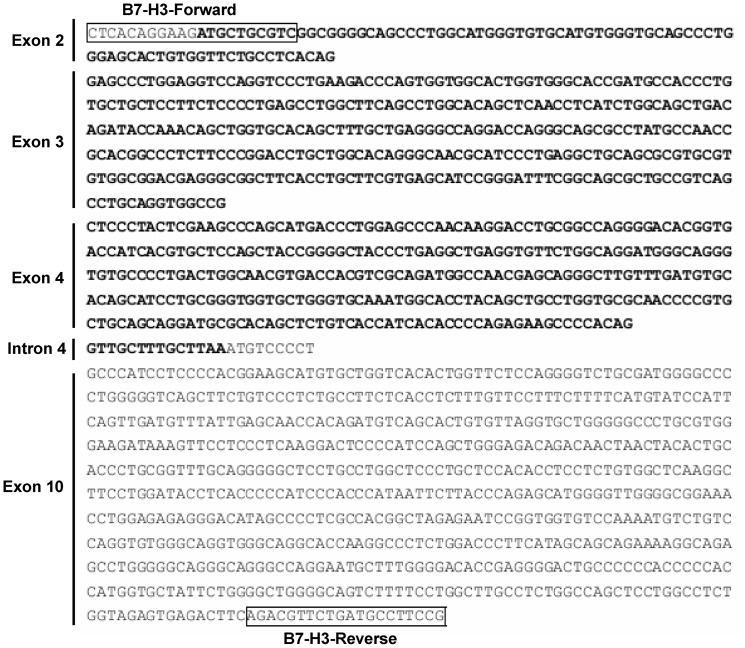
Nucleotide sequence analysis of spliced *sb7-h3* gene. The complete nucleotide sequence is shown. The boxed sequences are primer pair used to amplify *b7-h3* gene. The highlighted sequence is the ORF of spliced sB7-H3. The contained exons and intron are labeled on the left.

While amplifying human B7-H3 from the cDNA generated from U937 cells, we picked up a new isoform of B7-H3. Nucleotide sequence analysis showed the isoform lacked exon 5–9 in the protein-encoding sequence, but interestingly, retained 23 bp at the 5′ terminus of intron 4 and spliced out a 775 bp nucleotide sequence at the 5′ terminus of exon 10 in the cDNA (Figure 1). The isoform encodes a 248 amino acid length of protein, named spliced sB7-H3 here, which lacks a transmembrane domain and stops in the fragment of intron 4 after introducing an additional four amino residues at its C-terminus. Apart from U937 cell line, we were interested in the expression of the spliced sB7-H3 in hepatoma cell lines. cDNAs produced from a hepatoma cell line PLC/PRF/5 were submitted to amplifying spliced sB7-H3 using specific primer pair. A positive band was appeared in the result (Figure S1), and the amplified fragments were confirmed correctly to be spliced sB7-H3 by nucleotide sequencing. This result indicated spliced sB7-H3 is expressed in hepatoma cell lines.

### Spliced sB7-H3 generally exists in the PBMCs of healthy donors

**Figure 2:**
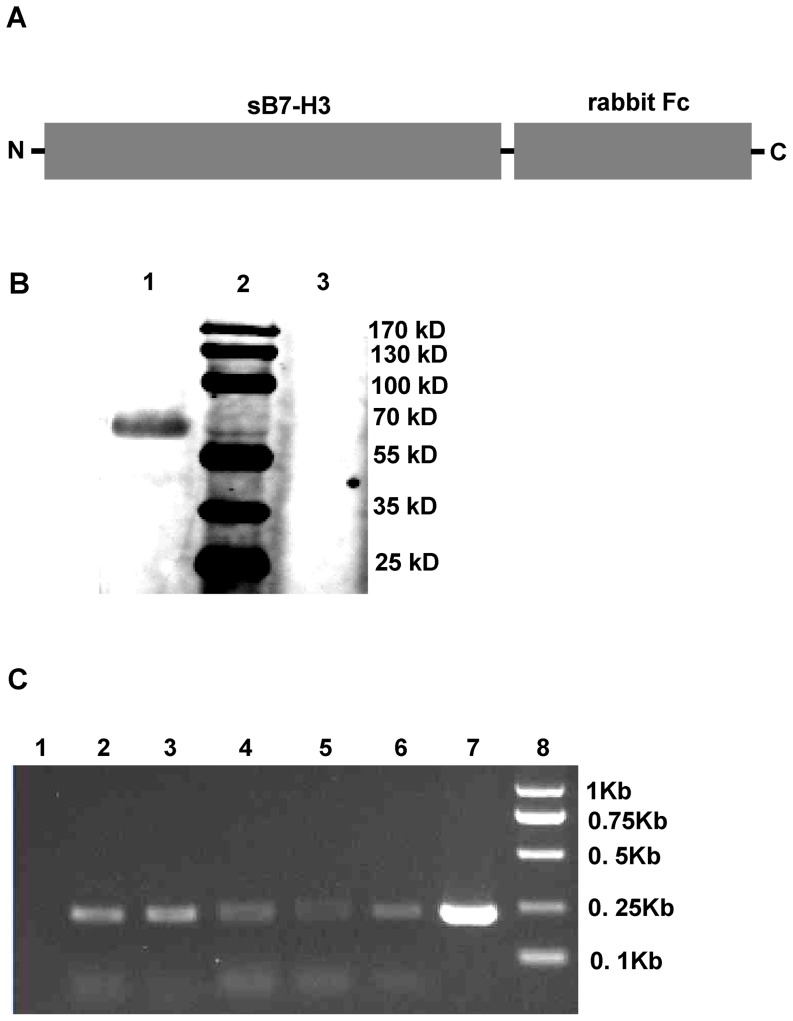
Spliced sB7-H3 is expressed as a soluble form and generally exists in healthy donors. A, The schematic diagram of chimeric sB7-H3-Fc: the complete spliced *sb7-h3* gene was fused with rabbit *fc* in one opened reading frame. B, pc-sB7-H3-Fc (lane 1) and vacant vector (lane 3) were transfected into 293T cells, respectively. The supernatant of transfected cells was harvested 24 h post transfection and submitted to western blot assay. Lane 2: prestained protein ladder. C, Investigation of spliced *sb7-h3* gene in the PBMCs of healthy donors: Lane 1: negative control; Lane 2–6: randomly selected healthy donors; Lane 7: positive control; Lane 8: DNA ladder.

To clarify the expression of spliced sB7-H3, we created a chimeric gene *sb7-h3-fc* by fusing rabbit Fc to the 3′-terminus of the spliced sB7-H3 in one ORF (Figure 2A). The chimeric gene was inserted into the pcDNA3.1(+) vector and transfected into 293T cells. After 48 h, the supernatant of transfected cells was subjected to pull-down and western-blot assays. As shown in figure 2B, pull-down assay detected sB7-H3-Fc in the supernatant of sB7-H3-Fc-transfected cells indicating that spliced sB7-H3 was expressed in soluble form and released from the cells as expected. As the expression of spliced sB7-H3 was confirmed in the U973 and PLC/PRF/5 cell lines, we would like to further determine whether it exists naturally in healthy PBMCs. We randomly selected five healthy donors to detect the cDNA of spliced sB7-H3 in PBMCs with PCR assay using the specific primer pair sB7-H3-F and sB7-H3-R. As shown in figure 2C, all samples were positive, indicating that spliced sB7-H3 is generally expressed in PBMCs from healthy donors.

### Spliced sB7-H3 retains the inhibitory function in T cell responses in vitro

**Figure 3:**
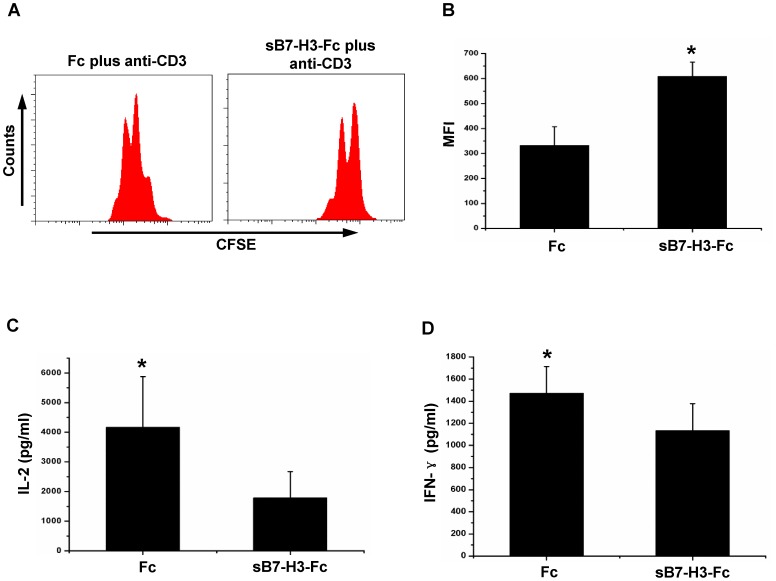
Functional analysis of spliced sB7-H3 on T cell response. CFSE-labeled purified T cells were seeded to a 96-well flat-bottomed plate precoated with 1 µg/ml anti-human CD3 (OKT3), plus either 10 µg/ml spliced sB7-H3-Fc or Fc. After culturing for 72 h, T cell proliferation and cytokines in the culture supernatant was analyzed. A, A representative plot of T cell proliferation analyzed with FACS assay. B, Statistical analysis of the mean fluorescence intensity (MFI) on T cells (mean ± s.e.m). C and D, the concentration of IL-2 and IFN-γ in the culture supernatant was analyzed with multiplex kit and analyzed by FlowCytomixPro software. “*” indicated P<0.05.

B7-H3 plays an inhibitory role in T cell response [11], [33]. The existence of an additional four amino residues introduced by intron 4 in spliced sB7-H3, compared to the ectodomain of 2Ig-B7-H3, led us to examine whether these additional amino residues affect the function of B7-H3 in T cell responses. We performed a stimulation assay in which isolated T cells were activated through TCR signaling in the presence of either sB7-H3-Fc or Fc protein. As shown in figure 3, upon activation through TCR signaling, T cell proliferation was significantly inhibited in the presence of spliced sB7-H3 compared to the Fc control group (P<0.05, Figure 3A and 3B). Evaluation of cytokine production in the culture supernatants found significantly reduced levels of both IL-2 and INF-γ in the presence of spliced sB7-H3 compared to the Fc control group (P<0.05, Figure 3C and 3D). These results revealed that spliced sB7-H3 has retained the inhibitory function in T cell responses.

### High level of sB7-H3 exist in sera of patients with HCC

**Figure 4:**
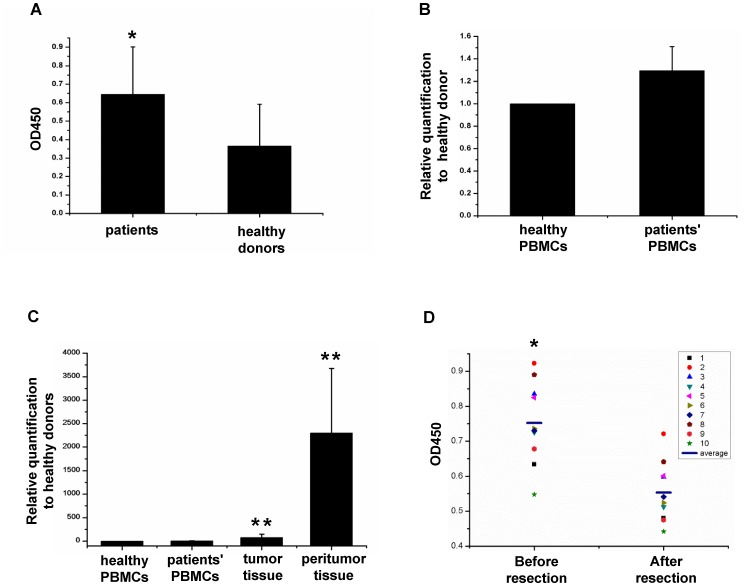
Comparison in the expression of spliced sB7-H3 between patients and healthy donors. A, Sera were obtained from healthy donors or patients with HCC. The expression level of spliced sB7-H3 was monitored with sandwich ELISA assay. B, The cDNAs produced from PBMCs of healthy donors or patients with HCC were analyzed for expression of spliced sB7-H3 by real-time PCR assay. A greater than 2-fold increase over health donors was considered clinical significant. C, The expression of spliced sB7-H3 in PBMCs of healthy donors and patients, as well as patients' tumor tissues and peritumor tissues were evaluated with comparative qPCR assay. A greater than 2-fold increase in value over healthy donors was considered clinical significant. D, Spliced sB7-H3 of individual serum from patients enrolled with HCC before and after resection was evaluated with sandwich ELISA assay. The horizontal line indicates the mean value of each group. “*” indicated P<0.05, “**” indicated P<0.01.

Previous research has shown that sB7-H3 in sera is higher in patients with carcinoma than in healthy controls [23], [25], [34], [35]. Based on this, we investigated whether spliced sB7-H3 would also be higher in the sera of HCC patients than in healthy individuals. As shown in figure 4A, the sandwich ELISA assay showed a significant difference between HCC patients and healthy donors (P<0.05), indicating that the concentration of spliced sB7-H3 in sera was higher in patients with HCC.

### Spliced sB7-H3 is predominantly found in hepatoma and peritumor tissues

We further determined the cause for the higher levels of spliced sB7-H3 in HCC patients' sera. It was hypothesized that the PBMCs from HCC patients express more spliced sB7-H3 than the healthy donors. To address this, we drew PBMCs from 25 HCC patients and 35 healthy donors and collected hepatoma and peritumor tissue samples through surgical resection. As shown in figure 4B, we found that the expression of spliced *sb7-h3* mRNA, as determined by relative quantification real-time PCR assay, was not significantly different in the PBMCs of HCC patients compared to healthy donors (P>0.05). In contrast, hepatoma and peritumor tissue samples showed significantly higher levels of spliced *sb7-h3* expression compared to PBMCs from either HCC patients (P<0.01) or healthy donors (P<0.05 and P<0.01, respectively), reaching up to 77 and 2300 times compared to the spliced *sb7-h3* in PBMCs from healthy donors, respectively (Figure 4C). It was interesting to note the expressed mRNA of spliced *sb7-h3* in peritumor tissues is higher than tumor tissues (P<0.01), reaching to more than 29 times (Figure 4C). These results indicated tumor and peritumor tissues produce more spliced *sb7-h3* than PBMCs. To further clarify this finding, 10 HCC patients were enrolled to assess the levels of spliced sB7-H3 in sera collected before hepatoma resection and 2 weeks after by ELISA assay. As shown in figure 4D, the spliced sB7-H3 was highly detected in sera before the surgical resection, but showed a significant decline two weeks after the resection (P<0.05). All above results further demonstrated that the higher levels of spliced sB7-H3 in patients' sera were due to the production by the hepatoma and peritumor tissues.

## Discussion

In this study, we identified a new isoform of B7-H3 in humans, spliced sB7-H3, which is spliced from the intron and encodes a soluble B7-H3 protein in sera. This new isoform appears to retain the inhibitory functions of B7-H3 on T cell responses despite the introduction of four additional amino residues in the sequence compared to the ectodomain of full-length 2Ig-B7-H3. Human B7-H3 has been known to exist as two isoforms, which are both expressed on the cell membrane and contain 2Ig and 4Ig like domains, respectively [8], [36], [37]. However, the reported functions of these two isoforms have been inconsistent, with some researches showing the costimulatory functions of B7-H3 in T cell responses [8], [9], [32], [38]–[40], and others revealing an inhibitory role in T cell responses [10]–[12], [33]. This inhibitory function of B7-H3 on T cell responses has led to further investigation into the association between B7-H3 expression and carcinoma progression [18], [25], [41], [42]. Results from these studies have indicated that the patients with strong B7-H3 expression on tumors were more likely to show disease spread. Apart from these two membrane B7-H3 isoforms, Xueguang Zhang and his colleagues first reported the existence of a sB7-H3 molecule in human cell lines and in the sera of healthy donors [22]. Moreover, sB7-H3 expression was found to correlate with disease progression in both cancer and HBV infection [23], [25], [34]. In relation with our results, which indicated that spliced sB7-H3 could inhibit T cell proliferation and reduce cytokine secretion, it is reasonable to speculate that high levels of spliced sB7-H3 in sera may also exacerbate cancer disease. By now, a number of members of the B7 superfamily have been shown to occur in sera [43]. These discoveries imply the ability of B7 superfamily molecules to exert these regulatory functions in even more fields through a soluble form in sera.

Previous study showed that soluble B7-H3 is produced from the release of membrane B7-H3 after digestion by matrix metalloproteinase (MMP) [22]. The supporting evidence was that matrix metalloproteinase inhibitor (MMPI) was able to enhance the expression of B7-H3 on the cell membrane [22], which was further supported by others [34]. For this study, we directly cloned the gene encoding sB7-H3 and showed through sequence analysis that it is produced after the introduction of additional sequences from intron 4 into the ectodomain of 2Ig-B7-H3 at the proximal membrane (figure 1). These results and the lack of direct evidence for MMP associated B7-H3 release from the membrane finds this theory to be unconvincing. Thus, contrary to previous reports [22], [34], our results revealed that production of soluble B7-H3 in sera occurs by alternative splicing of mRNA. Considering that it is impossible to generate a monoclonal antibody to differentiate spliced sB7-H3 from membrane B7-H3 because of only 4 amino acids discrepancy between them, we used real-time PCR assay with specific primer pair to analyze the spliced sB7-H3 in tissues at mRNA level, as shown in figure 4B and C, instead of mAb staining. This strategy ensures the specificity of the detection. Although a specific mAb against splice sB7-H3 is not available, it is convincible to use ELISA assay established with mAb against ectodomain of membrane B7-H3 to relatively quantify the spliced sB7-H3 in sera, because the sB7-H3 in sera is encoded by the spliced *sb7-h3* and the membrane form of B7-H3 resides on the cellular membrane and is not released into sera.

Researches show sB7-H3 to be higher in the serum/plasma of patients than those of healthy donors [25], [34], [35], however, where this increase comes from remains unknown. Our discovery of the spliced *sb7-h3* gene made it feasible to analyze the cause of this increase at the mRNA level. Relative qPCR results demonstrated that the increased levels of spliced sB7-H3 in patients' sera came from hepatoma and peritumor tissues and not from PBMCs (figure 4). Our finding perhaps presents a clue for explaining why sB7-H3 is higher in patients with other types of carcinomas [23], [25]. It is surprising to find that the expression of spliced sB7-H3 in peritumor tissues is higher than tumor tissues at mRNA level. Considering peritumor tissues are adjacent to tumor tissues, we speculated that the higher spliced sB7-H3 expression in peritumor tissues could perhaps be caused by the unknown factors which may be the interaction between the cancer and peritumor cells or some special components secreted by the hepatoma. It is may be a tumor protection mechanism, by which more T cell responses were inhibited, which allow accelerated tumor expansion. However, a further study is required to explore the underlying mechanism. Based on a previous study showing that infiltrated CD8^+^ T cells were exhausted in hepatoma tissues [44], we speculated that the increase of spliced sB7-H3 in the microenvironment of hepatoma and peritumor tissues leading to the inhibition of CD8^+^ T cells is at least a partial cause for this T cell exhaustion. We may also postulate that more rapid development of hepatomas occur from increased production of spliced sB7-H3 in the hepatoma and peritumor tissues. Therefore, our data may provide an explanation for why the level of sB7-H3 in serum is correlated with cancer progression.

In conclusion, we successfully cloned and identified a new spliced B7-H3 isoform that encodes the soluble B7-H3 protein. Our results showed that levels of soluble B7-H3 in serum were higher in patients with HCC than healthy controls. In particular, the increased expression of spliced sB7-H3 in tumor and peritumor tissues was directly correlated with the elevated serum sB7-H3 in the disease.

## Supporting Information

Figure S1 **Detection of the spliced sB7-H3 in PLC/PRF/5 cell line.** Total RNA of PLC/PRF/5 was extracted and submitted to produce cDNA. The spliced sB7-H3 expression was detected with PCR assay using specific primer pair sB7-H3-F/sB7-H3-R. Lane 1: PCR products from PLC/PRF/5 cell line. Lane 2: negative control. Lane 3: DL5000 DNA ladder. Lane 4: PCR products from the plasmid containing spliced sB7-H3.
(JPG)
