# Supplementary figures and images for: Characterization of a Soluble B7-H3 (sB7-H3) Spliced from the Intron and Analysis of sB7-H3 in the Sera of Patients with Hepatocellular Carcinoma
Source: PLoS One. 2013 Oct 23;8(10):e76965. doi: 10.1371/journal.pone.0076965 (PMC3806749; doi:10.1371/journal.pone.0076965)

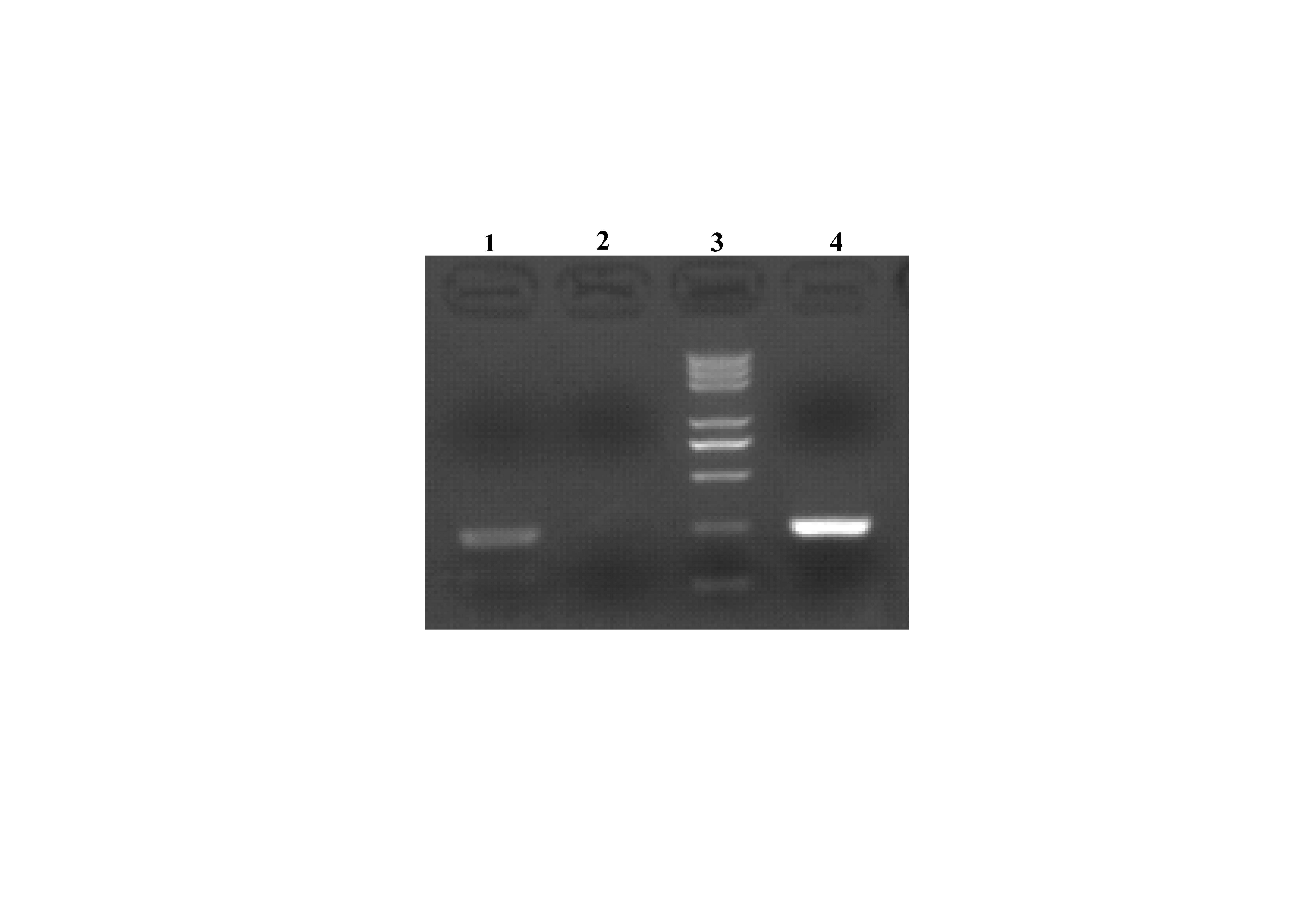

Supplement: Figure S1 — Detection of the spliced sB7-H3 in PLC/PRF/5 cell line. Total RNA of PLC/PRF/5 was extracted and submitted to produce cDNA. The spliced sB7-H3 expression was detected with PCR assay using specific primer pair sB7-H3-F/sB7-H3-R. Lane 1: PCR products from PLC/PRF/5 cell line. Lane 2: negative control. Lane 3: DL5000 DNA ladder. Lane 4: PCR products from the plasmid containing spliced sB7-H3. (JPG) [file pone.0076965.s001.jpg]
